# Small GSK-3 Inhibitor Shows Efficacy in a Motor Neuron Disease Murine Model Modulating Autophagy

**DOI:** 10.1371/journal.pone.0162723

**Published:** 2016-09-15

**Authors:** Estefanía de Munck, Valle Palomo, Emma Muñoz-Sáez, Daniel I. Perez, Begoña Gómez-Miguel, M. Teresa Solas, Carmen Gil, Ana Martínez, Rosa M. Arahuetes

**Affiliations:** 1 Departamento de Biología Animal II, Universidad Complutense de Madrid, Ciudad Universitaria, Madrid, Spain; 2 Centro de Investigaciones Biológicas-CSIC, Ramiro de Maetzu 9, Madrid, Spain; 3 Departamento de Bioquímica y Biología Molecular I, Universidad Complutense de Madrid, Ciudad Universitaria, Madrid, Spain; 4 Departamento de Biología Celular, Universidad Complutense de Madrid, Ciudad Universitaria, Madrid, Spain; University of Missouri Kansas City, UNITED STATES

## Abstract

Amyotrophic lateral sclerosis (ALS) is a progressive motor neuron degenerative disease that has no effective treatment up to date. Drug discovery tasks have been hampered due to the lack of knowledge in its molecular etiology together with the limited animal models for research. Recently, a motor neuron disease animal model has been developed using β-*N*-methylamino-*L*-alanine (L-BMAA), a neurotoxic amino acid related to the appearing of ALS. In the present work, the neuroprotective role of VP2.51, a small heterocyclic GSK-3 inhibitor, is analysed in this novel murine model together with the analysis of autophagy. VP2.51 daily administration for two weeks, starting the first day after L-BMAA treatment, leads to total recovery of neurological symptoms and prevents the activation of autophagic processes in rats. These results show that the L-BMAA murine model can be used to test the efficacy of new drugs. In addition, the results confirm the therapeutic potential of GSK-3 inhibitors, and specially VP2.51, for the disease-modifying future treatment of motor neuron disorders like ALS.

## Introduction

Amyotrophic lateral sclerosis (ALS) is a progressive neurodegenerative disease that affects nerve cells in brain and in the spinal cord. It is the most common motor neuron disease in adults, and causes progressive impairment of voluntary muscles involved in movement, respiration, speech, and swallowing. The progressive neuromuscular failure is invariably lethal within a few years after the onset of symptoms with some exceptions. The disease is divided in familial ALS (fALS), the hereditary form, that occurs only in 5%-10% of cases, and sporadic ALS (sALS), that accounts for the majority of the reported cases [1]. The economic and social burden of the disease is substantial [2], [3], and no effective treatment is currently available [4]. Recent findings in ALS molecular mechanisms could accelerate the discovery of an effective pharmacological intervention for this disease [5], and new therapeutic approaches are being explored uninterruptedly [6]. However, preclinical models used are based on the familial ALS mutation of superoxide dismutase SOD1 G93A, which represent a minority of the affected patients, and the use of an unrepresentative model could be the cause of drug failures in the clinic. Thus, there is an urgent need to find better models for this devastating disease.

In the last years, the neurotoxin β-N-methylamino-L-alanine (L-BMAA) has been proposed as one of the causes for the occurrence of sALS [7]. L-BMAA reached a great interest by its involvement in the origin of several neurodegenerative diseases [8] and its use in the search for sporadic animal models that emulate these pathologies [9]. This is of special importance for sALS where no established experimental models are currently available. Therefore, although there are many previous studies regarding L-BMAA toxicity [10], and adequate disease model for testing potential drugs has not achieved yet, and the SOD1 G93A transgenic mouse that mimic some fALS, representing minority of ALS cases, is the only ALS murine model used in drug discovery [11]. In previous work we have reported an experimental model of motor neuron disease in rats by their treatment with L-BMAA [12], [13], [14], that would allow not only for the study of the pathological neurodegenerative mechanism but also for the discovery of effective drugs that stop or delay the progression of this fatal disease.

In that model, animals treated with L-BMAA display a neurological impairment compatible with sALS symptoms. L-BMAA treatment starts at day 21 after birth and it is administered for 5 consecutive days. The onset of symptoms rapidly shows and the animals’ motor abilities gradually deteriorate.

L-BMAA treated animals present motor deficiencies together with ultrastructural alterations in both the endoplasmic reticulum and the mitochondria [12, 13, 15]. Molecularly, alterations of TAR DNA-binding protein 43 (TDP-43) and glycogen synthase kinase 3 (GSK-3), two major markers of neurodegenerative diseases, are found. Accumulation of TDP-43 is one of the main features of ALS and may have great relevance in the course of the disease [16].On the other hand, GSK-3 can play a key role in the signalling cascades leading to neuronal degeneration and its levels are reported to be increased in ALS patients [15].

GSK-3 is a serine/threonine kinase originally identified as a regulator of glycogen metabolism that plays a pivotal role in numerous cellular functions, including cell cycle regulation, differentiation and proliferation. Since the discovery of its involvement in the pathology of AD [17], GSK-3 has been proposed as a new target enzyme for neurodegenerative diseases or brain injury and, consequently, its inhibitors may represent new potential therapeutic drugs in neurodegenerative medicine [18].

GSK-3 is up-regulated in the brain and spinal cord of ALS patients [19] and the potential role of its inhibitors have recently been described [20]. In the last two years, reported data has supported the use of GSK-3 inhibitors as treatment for ALS and connected the post translational modifications found in TDP-43 with the upregulation of this kinase [21]. Efficacy of GSK-3 inhibitors both on stem-cell-derived motor neurons [22] and SOD1 G93A transgenic mice [23] have recently been reported, showing improved motor function and prolonged time until symptom onset. However, the efficacy of GSK-3 inhibitors in the most prevalent sporadic form of ALS remains yet to be studied.

Previous observations in animals treated with L-BMAA, such as the presence of fagosomes and damaged mitochondria in spinal cord motoneurons have led us to think that autophagic processes could be activated by this neurotoxin [13]. Zhang et al [24] described autophagic alterations in the SOD mutant mouse model, showing that there was a selective neurodegeneration of motoneurons and demonstrating that activation of the autophagy pathway accelerates the course of the disease and the neuropathological process. Autophagy has a key role in neurodegenerative diseases [25], both as damage protective mechanism as well as neuronal cell death mechanism [26]. ALS patients show an increment of autophagosomes in spinal cord motor neurons, that have also been noticed on rodent models [27–29]. Alterations of autophagic pathways have been extensively studied by several authors and have been proposed as a central mechanism of ALS pathogenesis [30–32]. Therefore, autophagy could be the leading cause for disease development in this model and its study could shed some light to the initial disease mechanism of sALS.

Our research group has great experience on the design and synthesis of GSK-3 inhibitors both as valuable pharmacological tools for biological studies and also as drug candidates [18, 33]. Among them, VP2.51 is a small heterocyclic compound that targets specifically GSK-3 and has already proved to be effective in multiple sclerosis model [34].

The main objective of the present study is to test the potential of our toxin-based motor neuron disease murine model as a pharmacological tool to be used in drug discovery, while analysing the neuroprotective action of the selected GSK-3 inhibitor. We have focused on the evaluation of neurological symptoms and on the analysis of the autophagy processes in motor neurons from motor cortex and spinal cord. The results show the recovery of the animals treated with VP2.51 immediately after finishing the L-BMAA treatment. Moreover, the treated group showed a significant decrease in autophagy in motor cortex. Altogether, these results represent the validation for the drug discovery process in a motor neuron disease mouse model with implications in the search for effective sALS treatments. To further confirm the involvement of GSK-3 in the neuroprotection observed in the animals, a cellular experiment was performed using a chemical genetic approach that utilizes chemically diverse GSK-3 inhibitors.

## Materials and Methods

Chemical reagents were bought from Sigma-Aldrich (St. Louis, MO). Melting point was determined with a Mettler Toledo MP70 apparatus. ^1^H NMR spectra were obtained on the Bruker AVANCE-300 spectrometer working at 300 MHz. Typical spectral parameters: spectral width 16 ppm, pulse width 9 μs (57°), data size 32 K. ^13^C NMR experiments were carried out on the Bruker AVANCE-300 spectrometer operating at 75 MHz. The acquisition parameters: spectral width 16 kHz, acquisition time 0.99 s, pulse width 9 μs (57°), data size 32 K. Chemical shifts are reported in values (ppm) relative to internal Me_4_Si and *J* values are reported in Hz.

Analyses indicated by the symbols of the elements were within ± 0.4% of the theoretical values. 1-(5-Acetyl-4-methyl-1,3-thiazol-2-yl)-3-(4-methoxybenzyl)urea (VP2.51): 4-methoxybenzyl isocyanate (0.874 ml, 6.12 mmol) in 40 mL of DMF is mixed with 5-acetyl-2-amino-4-methyl-1,3-thiazol (0.957 mg, 6.12 mmol) at 100°C for 14 h under nitrogen atmosphere and then cooled down to room temperature. AcOEt (50 mL) and H_2_O (50 mL) are added to the mixture. The organic extract is dried over anhydrous MgSO_4_ and evaporated under reduced pressure to yield a yellow solid. The solid is recrystallized in a mixture of MeOH/H_2_O to afford a white solid. Yield: 1.09 g, 56%. M.p. 196–197°C. ^1^H NMR (300 MHz, DMSO-d6): δ 10.85 (s, 1H, NH), 7.19 (d, *J* = 8.7 Hz, 2H, H_o_), 7.01 (bs, 1H, NH), 6.86 (d, *J* = 8.7 Hz, 2H, H_m_), 4.22 (d, *J* = 5.9 Hz, 2H, (CH_2_-NH)), 3.69 (s, 3H, OMe), 2.47 (s, 3H, CH_3_-thiazole), 2.39 (s, 3H, CH_3_-CO). ^13^C NMR (75 MHz, DMSO-d6): δ 190.2 (CO), 161.7 (NHCONH), 158.3 (C_ar_-O), 153.2 (C2thiazole), 132.9 (C4thiazole), 131.1 (C_i_), 128.7 (C_o_), 128.3 (C5thiazole), 113.8 (C_m_), 113.6 (C_m_), 55.1 (OMe), 42.5 (CH_2_-NH), 29.9 (CH_3_-CO), 18.1 (CH_3_-thiazole). HPLC: C18, 3.5 μM, 4.6 x 50 mm column, H_2_O/CH_3_CN 10:100 gradient in 5 min. Purity > 99%, r.t. = 4.08 min. MS (ESI+): m/z 320 [M+1]. Elemental analysis of VP2.51 is reported on S1 Table.

### Inhibition of GSK-3

Human recombinant GSK-3β was purchased from Millipore (Millipore Iberica S.A.U.). The prephosphorylated polypeptide substrate was purchased from Millipore (Millipore Iberica S.A.U.). Kinase-Glo Luminescent Kinase Assay was obtained from Promega (Promega Biotech Ibérica, SL). ATP and all other reagents were from Sigma-Aldrich (St. Louis, MO). Assay buffer contained 50 mM HEPES (pH 7.5), 1 mM EDTA, 1 mM EGTA, and 15 mM magnesium acetate.

The method of Baki et al [35] was followed to analyse the inhibition of GSK-3β. Kinase-Glo assays were performed in assay buffer using black 96-well plates. In a typical assay, 10 μl (10 μM) of test compound (dissolved in dimethyl sulfoxide (DMSO) at 1 mM concentration and diluted in advance in assay buffer to the desired concentration) and 10 μl (20 ng) of enzyme were added to each well followed by 20 μl of assay buffer containing 25 μM substrate and 1 μM ATP. The final DMSO concentration in the reaction mixture did not exceed 1%. After 30 min incubation at 30°C the enzymatic reaction was stopped with 40 μl of Kinase-Glo reagent. Glo-type luminescence was recorded after 10 min using a FLUOstar Optima (BMG Labtechnologies GmbH, Offenburg, Germany) multimode reader. The activity is proportional to the difference of the total and consumed ATP. The inhibitory activities were calculated on the basis of maximal activities measured in the absence of inhibitor. The IC_50_ was defined as the concentration of each compound that reduces by 50% the enzymatic activity. To investigate the inhibitory mechanism of VP2.51 on GSK-3β, a kinetic study varying both ATP (from 1 to 50 μM) and VP.251 (from 0.5 to 1 μM) concentrations were performed using the ADP-Glo Kinase Assay [36].

To study the type of enzymatic inhibition for the compounds, measurements after several times of incubation of the enzyme with the inhibitor VP2.51 were performed. A reversible inhibitor does not increase the inhibition of the enzyme with the time of incubation, while an irreversible inhibitor increases the inhibition percentage as the time of incubation with the enzyme increases.

### *In vitro* parallel artificial membrane permeability assay (PAMPA)

Prediction of the brain penetration was evaluated using a parallel artificial membrane permeability assay (PAMPA). Ten commercial drugs, phosphate buffer saline solution at pH 7.4 (PBS), DMSO and dodecane were purchased from Sigma, Across organics, Aldrich and Fluka. The porcine polar brain lipid (PBL) (catalog no. 141101) was from Avanti Polar Lipids. The donor plate was a 96-well filtrate plate (Multiscreen IP Sterile Plate PDVF membrane, pore size is 0.45 μM, catalog no. MAIPS4510) and the acceptor plate was an indented 96-well plate (Multiscreen, catalog no. MAMCS9610) both from Millipore. Filter PDVF membrane units (diameter 30 mm, pore size 0.45 μm) from Symta were used to filter the samples. A 96-well plate UV reader (Thermoscientific, Multiskan spectrum) was used for the UV measurements. Test compounds were dissolved in DMSO (250 μL). 25 μL of this compound stock solution was taken and 225 μL of DMSO and 4750 μL of PBS pH 7.4 buffer were added to reach 5% of DMSO concentration in the experiment. These solutions were filtered. The acceptor 96-well microplate was filled with 180 μL of PBS:DMSO (95:5). The donor 96-well plate was coated with 4 μL of porcine brain lipid in dodecane (20 mg mL^-1^) and after 5 minutes, 180 μL of each compound solution was added. 1–2 mg of VP2.51 was dissolved in 250 μL of DMSO and 4750 μL of PBS pH 7.4 buffer, filtered and then added to the donor 96-well plate. Then the donor plate was carefully put on the acceptor plate to form a “sandwich”, which was left undisturbed for 4 h at 25°C. During this time the compounds diffused from the donor plate through the brain lipid membrane into the acceptor plate. After incubation, the donor plate was removed. The concentration of VP2.51 and commercial drugs in the acceptor and the donor wells was determined by UV plate reader. Every sample was analyzed at three to five wavelengths, in 3 wells and in three independent runs. Results are given as the mean [standard deviation (SD)] and the average of the three runs is reported. Validation was made comparing the reported permeabilities values of commercial drugs with the experimental data obtained employing this methodology. A good correlation between experimental-described values was obtained Pe (exptl) = 1.0123 (bibl) + 0.1038 (R^2^ = 0.975) (S1 Fig). From this equation and following the pattern established in the literature for BBB permeation prediction [37] we could classify compounds as CNS + when they present a permeability > 4.15 x 10^−6^ cm s^-1^ (S2 Table).

### Cell culture

SH-SY5Y cells on a 96 well plate ere incubated with L-BMAA (10 mM) with or without inhibitors (10 μM). A control experiment was set at the same time without L-BMAA or inhibitors. After 24 h a MTT was performed to determine cell viability using known procedures in the literature. [38] Experiment was performed in triplicate.

### Animals

Assays were carried out using male rats of *Rattus norvegicus*, albino variety, Wistar strain, that were supplied by Harlan Interfauna Ibérica S.A. (Barcelona, Spain). Animals were kept at constant temperature (21 ± 1°C) on a reversed light/dark cycle with diets and water *ad libitum* (A04 commercial rodent diet, Panlab, Barcelona, Spain). The trials were approved by the Ethics Committee for Animal Experimentation of the Universidad Complutense de Madrid according to EU Directive 2010/63/EU for animal experiments. Animal’s health was monitored by weight control. No animals were severely ill or died at any time prior to the experimental endpoint. Animals were sacrificed at the established time point under general anaesthesia with isoflurane (ISOFLO®, Abbott Laboratories, Abbott Park, IL, USA).

### L-BMAA treatment

At weaning time (postnatal day 21) animals, with a weight between 45 and 55 g, were intraperitoneally (i.p.) injected with 300 mg/kg of L-BMAA (L-BMAA hydrochloride, Sigma-Aldrich, Ref. B107), during 5 consecutive days. The injected solution was composed by 50 mg of L-BMAA dissolved in 500 μl of PBS (0.1 mg / μl). Controls were injected with a similar volume of PBS, according to their weight.

### VP2.51 treatment

VP2.51 was dissolved in a stock solution of DMSO (dimethyl sulfoxide, Sigma) at 100 mg/mL. This stock was used to prepare a final solution of 1 mg/mL in PBS with 5% DMSO and 5% Tween® 80 (Sigma). Rats were injected i.p. 2.5 mg/Kg beginning at two different times: on the first day after treatment with L-BMAA (PT1) and on day post-treatment 30 (PT30). Injection treatment was performed for 15 consecutive days. Two control groups that had not received the L-BMAA treatment were injected with VP2.51 at the same scheduled times of the L-BMAA and VP2.51 treated groups.

In summary, 6 different groups of 12 animals each were used: Control (only PBS injected), L-BMAA, L-BMAA+VP2.51 PT1, VP2.51 PT1, L-BMAA+VP2.51 PT30, VP2.51 PT30. Weight monitoring was performed every day during 100 days. After this time the animals were sacrificed.

### Neurological evaluation

The neurological tests were carried out blindly as described by de Munck et al [13], once a week during 100 days. Briefly, the neurological assess was carried out with three different tests: ambulation, tail suspension test and strength test. The three approaches of the assessment had not an equal contribution to the final neurological evaluation: the tail suspension test represents 70% of the total assessment, and the other two tests were used to fine tune the details of the value with an importance of 15% each. During the ambulation both postural control and the way the hind limbs were leant during motion was observed. In the tail suspension test, the rat was suspended by the tail at 10 cm over the surface. The ability to keep the hind limbs in the normal neutral position adopted by rodents in such situation, i.e., describing a “T” was evaluated, together with the stiffness of the limbs and the inter-limb coordination during kicking. The strength test and was carried out setting down the rats on a flat surface, both forelegs and hind limbs on it, while pulling the tail softly to the opposite direction of animal usual move course. The ability the rats had to drag and offer resistance, assessing their faculty to kept hind limbs under the body without spreading their legs away from it was observed. The neurological evaluation was established in a scale comprised between 0 and 10, where 0 is the evaluation given to animals which are not symptomatic, control animals, and 10 belongs to a state of total functional loss of the hindlimbs and postural control. Three videos showing the tests performed for the neurological evaluation of control (S1 Movie), L-BMAA (S2 Movie) and L-BMAA +VP2.51 (PT1) (S3 Movie) rats can be found in the SI.

### Analysis of LC3-II and P-mTOR

The animals were sacrificed under general anaesthesia with isoflurane (ISOFLO®, Abbott Laboratories, Abbott Park, IL, USA), 5% O_2_. The entire brain and the lumbar spinal cord were rapidly removed and stored at -80°C.

To analyse LC3-II and P-mTOR by western-blot, 100 mg of motor cortex and lumbar spinal cord of each animal were homogenized in a RIPA buffer: 50 mM Tris–HCl pH 7.4, 50 mM NaCl, Nonidet P-40 1%, 1 mM EDTA, 1 mM PMSF, 1 mg/mL aprotinin, 1 mg/mg leupeptin, 1 mg/mL pepstatin, at a rate of 10 mL of buffer per 1 mg of tissue. The mixture was sonicated for 2 min with 2 s pulses of sonication and 2 s off at amplitude of 40%. Once the samples were homogenized, the evaluation and protein electrophoresis was performed. The protocol was adapted from Zhang et al [24] as described in Muñoz-Sáez et al [14].

Before the electrophoresis, the protein concentrations of all samples were determined by Bradford assay (Sigma–Aldrich Inc., St. Louis, MO, USA; Bradford, 1976).

Electrophoresis for protein separation was performed by SDS-PAGE. In all cases, the concentrating gel contained 6% polyacrylamide. Regarding LC3-II, the percentage of polyacrylamide was 12% for the separating gel and for P-mTOR an 8% polyacrylamide separating gel was used. Subsequently, proteins from the gels were transferred to a nitrocellulose membrane (AmershamTM HybondTM–ECL) in a wet system at 10 V o/n and 4°C. Membranes were incubated with following primary antibodies: a rabbit polyclonal anti-tubulin β-III (Novus Biologicals, Littleton, CO, USA), a rabbit polyclonal anti-LC3-II (Novus Biologicals, Littleton, CO, USA) and rabbit polyclonal anti-mTOR (Phospho-Ser2448) (Novus Biologicals, Littleton, CO, USA). As a secondary antibody, we applied Donkey anti-rabbit IgG-Fc Horseradish Peroxidase (HRP) (Bethyl Laboratories, Montgomery, TX, USA). All the antibodies were used according to the manufacturers’ specifications. Proteins were detected by enhanced chemiluminescence. Digital images were acquired using a Fujifilm Intelligent Darkbox II (Fuji Systems USA, Stamford, CT) and the image processing for quantification was conducted with free image processing software Image J.

### Statistical analysis

Results are presented as means ± SEM in graphics. Data were adjusted to the most suitable regression model, weight by Boltzmann sigmoidal regression and neurological evaluation, after normalisation, by linear regression, and then analysed by F-test. Data from each biochemical experiment was analysed separately and treated by ANOVA including a Bonferroni post-test. Student’s t-test was also used to compare the samples. Experiments were repeated three times with triplicate samples for each experiment. The data were analysed using Graph Pad Prism v5.03 software. Values of p ≤ 0.05 were considered statistically significant.

## Results

### Characterization of VP2.51 as a brain permeable GSK-3 inhibitor

Several authors have recently highlighted the potential of GSK-3 inhibitors to restore or reduce damage to the central nervous system caused by neurodegenerative diseases or other factors [39]. Genetic or pharmacological inhibition of GSK-3 protects neurons from a wide range of environmental stresses including hypoxia and amyloid toxicity, which may be relevant for the treatment of stroke or diseases such as Alzheimer’s disease [40]. The involvement of GSK-3 in ALS has recently been reviewed in various experimental studies where GSK-3 inhibitors were used in animal and cell models [41], [42]. Clinical studies have also shown the potential therapeutic role of these molecules [20].

To assess the therapeutic potential of GSK-3 inhibitors in a motor neuron disease murine model the 1,3-thiazole derivative named VP2.51 was selected. This heterocyclic molecule was designed based on the crystal structure of the complex 1Q5K by modification and elimination of toxicophores residues such the nitro functional group changed by chemical groups that allow switching from reversible to irreversible inhibitors [43], [44].

**Fig 1:**
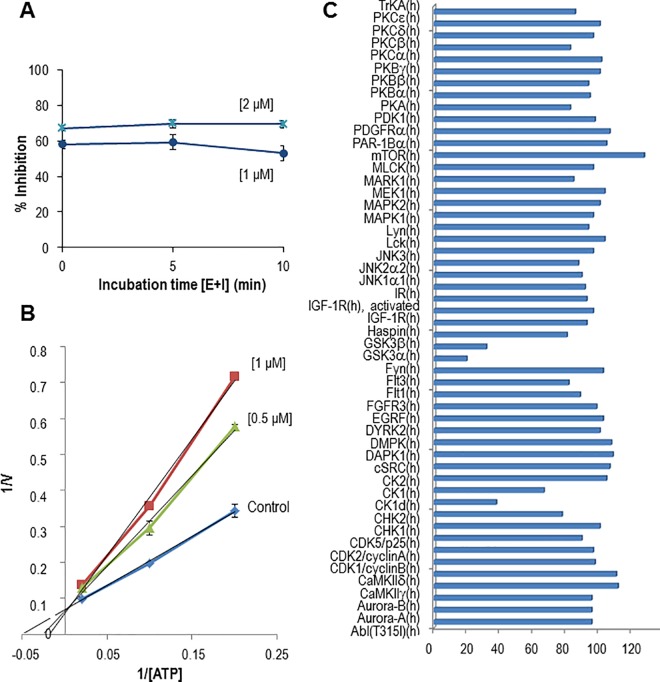
Biological activity of VP2.51 on GSK-3 and other protein kinases. A) Time-dependent GSK-3 inhibition of VP2.51 at 1 and 2 μM. B) Kinetic GSK-3 inhibition data determined for VP2.51. ATP concentrations in the reaction mixture varied from 1 to 50 μM. Compound concentrations used are depicted in the plot, and the concentration of GS-2 was kept constant at 12.5 μM. Each point is the mean of two different experiments, each one analyzed in duplicate. C) Kinase profiling of VP2.51 on human recombinant kinases. The percentage (%) of kinase activity after the treatment with a **fixed concentration (10 μM) of the compound is shown.**

Its GSK-3 inhibitory activity has an *in vitro* IC_50_ value of 0.62 ± 0.15 μM on human recombinant enzyme, and time dependent experiments showed a reversible inhibition (Fig 1A). Kinetic experiments were also performed showing that VP2.51 competes *in vitro* with ATP for the GSK-3 binding site (Fig 1B). As ATP-competitive inhibitors may have selectivity *in vivo* complications, a kinase profiling over a panel of fifty different human kinases was done using a fixed concentration of VP2.51 at 10 μM. VP2.51 shows a high selectivity profile targeting only both isoforms of GSK-3, α and β, and with less potency, casein kinase-1δ (CK-1δ) (Fig 1C). Further determination of the IC_50_ value for CK-1δ inhibition following a described protocol, showed a value of 7.28 ± 0.87 μM, tenfold greater than the corresponding to GSK-3 inhibition. It is worth mentioning that CK-1δ inhibitors have recently been reported as new drug candidates for ALS therapy based on their ability to reduce TDP-43 hyperphosphorylation [44].

Finally, the ability to cross the blood-brain barrier (BBB) was determined. This is a key feature for considering this compound as a drug candidate for the potential treatment of central nervous system diseases. VP2.51 permeability through the BBB was evaluated by a parallel artificial membrane permeability assay (PAMPA) using porcine brain membrane lipid and ten commercial drugs following the methodology described by Di et al. [37] Its effective permeability was 7.6 ± 0.4 x 10^−6^ cm/s (S1 Fig and S2 Table), which is compatible with a good brain penetration. Lastly, it is worth mentioning that VP2.51 is a weak or partial inhibitor of the cytochromes P450 family [45] which points to the safe *in vivo* profile of this compound.

### L-BMAA and VP2.51 treatment

At weaning time, animals were intraperitoneally (i.p.) injected with 300 mg/kg of L-BMAA, during 5 consecutive days. After neurotoxin treatment, VP2.51 was injected i.p. 2.5 mg/Kg beginning at two different times: on the first day after treatment with L-BMAA (PT1) and on day post-treatment 30 (PT30). Injection treatment was performed for 15 consecutive days. Two control groups that had not received the L-BMAA treatment were injected with VP2.51 at the same scheduled times of the L-BMAA and VP2.51 treated groups.

Collectively 6 different groups were used: control (only PBS injected), L-BMAA, L-BMAA+VP2.51 PT1, VP2.51 PT1, L-BMAA+VP2.51 PT30, VP2.51 PT30 and they were observed during 100 days.

### Evaluation of disease progression on motor neuron disease model

Weight progression, neurological evaluation and analysis of autophagy were performed blindly to assess the disease progression on the rats.

#### Weight progression

**Fig 2:**
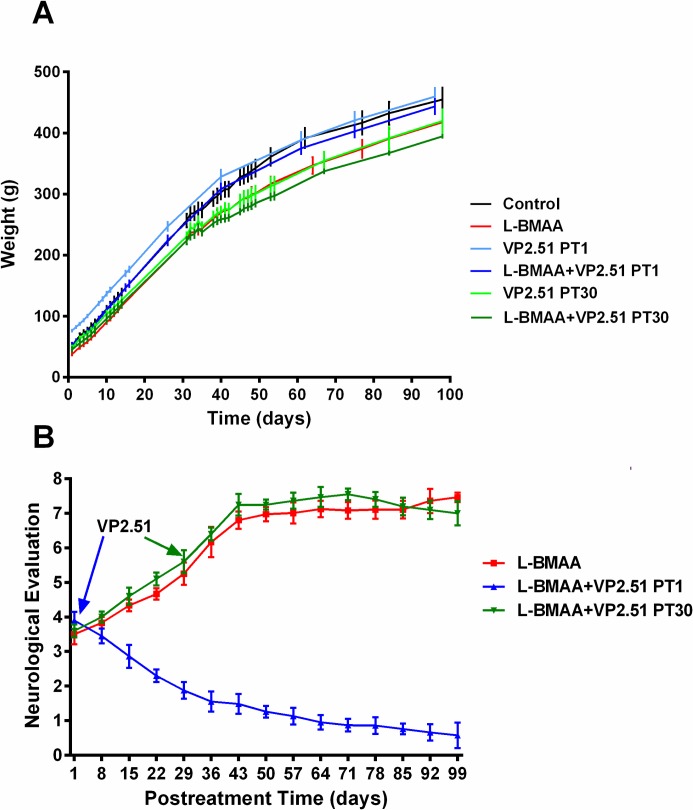
Weight progression and neurological evaluation. A) Evolution of rat weight of different treatment groups (12 animals in each group): control, L-BMAA (300 mg/kg/day during 5 consecutive days treated at weaning), VP2.51 (2,5 mg/kg during 15 days) with a group treated at PT1 and another at PT30, L-BMAA (with the same treatment as the group of L-BMAA) + VP2.51 (with the same treatment groups VP2.51) with a group treated at PT1 and another at PT30. B). Evolution of neurological evaluation in the treatment assay with VP2.51 (12 animals in each group). The control group and the groups treated exclusively with VP2.51 (at PT1 and PT30) are not shown because its assessment coincides with the horizontal axis. The time when the inhibitor is added is indicated with arrows.

Statistical analysis revealed no significant differences between groups, indicating that VP2.51 and L-BMAA do not affect physical development of rats neither alone or co-administered (Fig 2). These results show that VP2.51 alone does not cause deleterious physical side effects in animals.

#### Neurological evaluation

A scale comprised between zero and 10 points, where zero is the evaluation given to animals which are not symptomatic, control animals, and 10 score belongs to a state of total functional loss of the hind limbs and postural control was used to evaluate neurological abnormalities.

**Fig 3:**
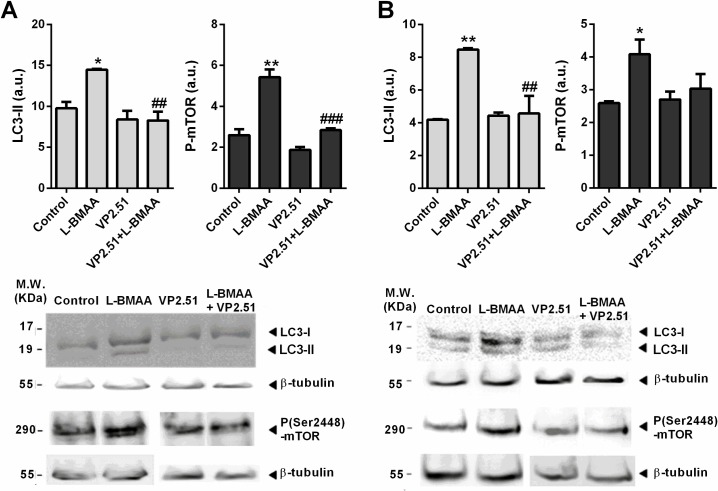
Autophagy evaluation in motor cortex and lumbatr spinal cord A) Analysis of LC3-II and P-mTOR by western-blot in the motor cortex of control and treated animals. Proteins were detected by Western blotting using β-tubulin for calibration of sample loading. The protein bands were quantified by Image Multigauge v3.0 software. Immunoblots are representative of one of three experiments with similar results. These results were quantitated in arbitrary units and were represented in the accompanying graphs as mean ± SEM. *, ** Indicate differences of p-value <0.05 <0.01 with the control group, respectively. #, ### indicate differences of p-value <0.05 <0.001 with the group of animals treated with L-BMAA, respectively. B) Analysis of LC3-II and P-mTOR by western-blot in the lumbar spinal cord of control and treated animals. Proteins were detected by Western blotting using β-tubulin for calibration of sample loading. The protein bands were quantified by Image Multigauge v3.0 software. Immunoblots are representative of one of three experiments with similar results. These results were quantitated in arbitrary units and were represented in the accompanying graphs as mean ± SEM. *, *** Indicate differences of p-value <0.05 <0.001 with the control group, respectively. ## Indicate differences of p-value <0.01 with the group of animals treated with L-BMAA.

L-BMAA treated animals reached a value of 7 points in the neurological evaluation score. The animals show a major impediment to maintain a correct posture while the tail suspension test was carried out. In addition, they suffer from a lateralized ambulation, and have problems completing turns on the march. Further, the animals kept the legs relaxed and pitched towards the ventral side. Concerning the strength test, the treated rats’ hind limbs protruded from the vertical projection of the body on the table while they sought to counteract the pressure on their tails (S2 Movie) [13]. The group treated with VP2.51 from day 1 after treatment with L-BMAA (L-BMAA + VP2.51 PT1) (S3 Movie) presented highly significant differences in the evolution of the neurological evaluation from the group treated only with L-BMAA (F = 89.34, p <0.0001) (Fig 3). These animals markedly improved their neurological evaluation, almost reaching levels of control animals in three months. These animals maintain a correct posture at the tail suspension test and show neither lateralized ambulation nor strength loss. However, animals treated with VP2.51 from day 30 post-treatment after injections with L-BMAA (L-BMAA + VP2.51 PT30), did not show significant differences with the animals treated with L-BMAA.

The control group (S1 Movie) and the groups treated exclusively with VP2.51 (PT1 and PT30) had a neurological evaluation score of zero and are not shown in Fig 2. The results obtained from the neurological evaluation confirm that VP2.51 does not promote either neurological pathologies or observable toxicities and clearly has a neuroprotective effect when administered during two weeks from the first day after treatment with L-BMAA (PT1). Neurological symptoms of treated animals improved markedly to almost the level of controls within three months.

#### Analysis of Autophagy

As VP2.51 prevents the neurological effects of L-BMAA in neurological evaluation of animals when administered from day PT1, we analyzed the possible effects of this GSK-3 inhibitor on autophagy caused by L-BMAA in the motor cortex and lumbar spinal cord. LC3-II is used as a standard marker of autophagy levels in tissues [46], and was analysed by Western blotting. Our results indicate that animals treated with L-BMAA present both LC3-II and P-mTOR levels significantly higher than control animals in the motor cortex (LC3-II: t = 5.969; p = 0.027; P-mTOR: t = 5.889, p = 0.0042) (Fig 3A) and the lumbar spinal cord (LC3-II: t = 46.85; p = 0.0005; P-mTOR: t = 3.257, p = 0.031) (Fig 3B). ANOVA test showed statistical differences between the different groups regarding LC3-II (F = 5.56, p = 0.0234) and P-mTOR (F = 37.22, p <0.0001) in the motor cortex. Moreover, animals treated with L-BMAA+VP2.51 at PT1 presented both LC3-II levels (t = 3.547, p <0.05) and P-mTOR levels (t = 7.223, p <0.001) significantly lower than animals treated with L-BMAA.

LC3-II levels in the lumbar spinal cord offered significant differences between groups (F = 13.12, p = 0.0019), being higher in animals treated with L-BMAA and lower in controls and L-BMAA +VP2.51 to PT1 treated animals. (t = 5.083, p <0.01). Animals treated with L-BMAA + VP2.51 to PT1 show a lower non-significant P-mTOR level in this tissue compared with L-BMAA treated groups.

#### Cell culture

**Fig 4:**
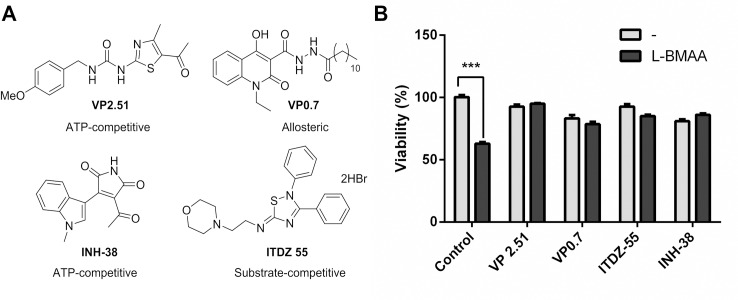
Neuroprotection of cellular L-BMAA model by diverse GSK-3 inhibitors. A) Diverse GSK-3 inhibitors selected showing their inhibition kind. B) Cell viability measured in the experiment with or without inhibitors.

Mechanistically and structurally diverse GSK-3 inhibitors were tested in a cellular model of L-BMAA to study whether GSK-3 inhibition was implicated in neuroprotection at L-BMAA damage, using a forward chemical genetic approach. VP2.51 and INH-38 [43] are ATP-competitive inhibitors of GSK-3, ITDZ 55 [47] is a substrate competitive inhibitor and VP0.7[48] is an allosteric inhibitor that does not compete with the ATP nor the substrate in its GSK-3 inhibition and was proposed to bind to a pocket at the C-lobe of the enzyme. Cells were incubated with L-BMAA together with or without inhibitors for 24 h and cell viability was determined. Fig 4 shows how GSK-3 inhibitors protect cells from L-BMAA damage, engaging GSK-3 as the target for the observed effect.

## Discussion

The finding of the neurotoxic amino acid L-BMAA in Alzheimer’s disease and ALS patients has led some authors to propose the hypothesis that L-BMAA accumulation could be related to the development of several neurodegenerative diseases [49]. L-BMAA is an unnatural amino acid that can become part of protein chains. Once incorporated into the chain, proteins can no longer fold properly. Accumulation of L-BMAA in the proteins of nerve cells, which need to last a lifetime, would provide a mechanism of how the toxin could have effects in later times. Neurons accumulate damaged proteins over time, and once they reach a critical level, it causes the cell to undergo apoptosis. In this regard, studies in our laboratory have shown that L-BMAA treated rats would be a good experimental model of a motor neuron disease mimicking some aspects of sALS [13] and we have applied it to study the therapeutic action of a GSK-3 inhibitor for the treatment of this neurodegenerative disorder.

The results obtained from the neurological evaluation could be considered as clinically relevant since the administration of a drug prior to symptoms onset allows a protective therapy. In this case, the GSK-3 inhibitor used has shown neuroprotective activity against neurotoxicity caused by L-BMAA. As the compound administration starts after the induction of ALS symptoms by 5 days treatment with L-BMAA, the efficacy shown with VP2.51 may be translated into a potential clinical treatment for early diagnosed patients: as soon as the first neurological sign is detected in a patient or in case a genetic analysis identified a familiar ALS, the treatment with VP2.51 or any other GSK-3 inhibitor may be recommended. Results also show that the earlier the disease is diagnosed, the better response to the treatment is to be expected.

Results from the autophagy analysis show that L-BMAA causes activation of autophagic processes and are in agreement with those obtained by Hetz et al [50] in spinal cord samples of sALS and fALS patients, where they observed a marked induction of LC3-II. The importance of apoptosis in diseases characterized by proteinopathies, such as ALS has been emphasized recently [51, 52]. Thanks to research in this direction it has been established that high levels of protein aggregates can cause dysfunction of the unfolded protein response [53], and subsequent compensatory activation of autophagy [54].

In the present work it is shown how VP2.51 prevents the activation of autophagic processes in L-BMAA treated animals when it is administered from day 1 post treatment. In motor cortex, VP2.51 causes the decrease of the marker protein autophagy LC3-II and the concomitant decrease of P-mTOR respect to L-BMAA treated group, reaching levels similar to those obtained in the control group. In lumbar spinal cord, VP2.51 administration to PT1 after treatment with L-BMAA also seems to prevent activation of autophagic processes, given the reduction in LC3-II levels found. Although in this case a significant decrease in P-mTOR levels respect to the group treated only with L-BMAA was not shown, a tendency to decrease can be noticed. Therefore, VP2.51 treatment prevents activation of autophagy produced by L-BMAA in the motor cortex and the lumbar spinal cord, two of the main motor control centres affected by ALS.

Finally, a cellular experiment was performed with diverse GSK-3 inhibitors to study target engagement in neuroprotection at the event of L-BMAA damage. Using a forward chemical genetic approach mechanistically and structurally varied GSK-3 inhibitors showed an increased cell viability when coincubated with L-BMAA, thus confirming the involvement of GSK-3 as a key player of cell protection in this model.

## Conclusions

In the present work the pharmacological utility of a motor neuron disease murine model based on the L-BMAA neurotoxicity was evaluated showing that treatment with VP2.51, a selective GSK-3 inhibitor, from the earliest moments of L-BMAA action, prevents the onset of motor symptoms produced by this neurotoxic amino acid. In addition, this neuroprotective effect of VP2.51 is associated to inhibition of autophagy in motor cortex and lumbar spinal cord. We show how autophagy may be playing a key role in disease progression and how its suppression may be fundamental to reduce or reverse disease pathology. The neuroprotective effect of VP2.51 this murine model is a promising finding and requires further study in order to clarify its action. An additional cellular experiment was performed with mechanistically and structurally distinct GSK-3 inhibitors showing the involvement of the target in this motor neuuron disease model induced by L-BMAA. GSK-3 inhibitors and specifically VP2.51 emerge as a potential therapeutic option for the early diagnosed treatment of motor neuron diseases like ALS.

## Supporting Information

S1 Fig Linear correlation between experimental and reported permeability of commercial drugs using in the PAMPA-BBB assay. (DOCX)

S1 Movie Control group. (RAR)

S2 Movie L-BMAA treated group. (RAR)

S3 Movie VP2.51 (PT1) and L-BMAA treated group. (RAR)

S1 Table Elemental analysis of VP2.51. (DOCX)

S2 Table Permeability in the PAMPA-BBB assay for 10 commercial drugs and VP2.51 with its predictive penetration in the CNS. (DOCX)

## Abbreviations

ALS: amyotrophic lateral sclerosis
sALS: sporadic amyotrophic lateral sclerosis
L-BMAA: β-N-methylamino-L-alanine
GSK-3: glycogen synthase kinase 3
BBB: blood brain barrier
LC3-II: microtubule-associated protein light chain 3
mTOR: mammalian target of rapamycin
