# Supplementary material for: Small GSK-3 Inhibitor Shows Efficacy in a Motor Neuron Disease Murine Model Modulating Autophagy
Source: PLoS One. 2016 Sep 15;11(9):e0162723. doi: 10.1371/journal.pone.0162723 (PMC5025054; doi:10.1371/journal.pone.0162723)
Supplement: S1 Fig — (DOCX) [file pone.0162723.s001.docx]

**Figure S1.** Linear correlation between experimental and reported permeability of commercial drugs using in the PAMPA-BBB assay.
