# Supplementary material for: Small GSK-3 Inhibitor Shows Efficacy in a Motor Neuron Disease Murine Model Modulating Autophagy
Source: PLoS One. 2016 Sep 15;11(9):e0162723. doi: 10.1371/journal.pone.0162723 (PMC5025054; doi:10.1371/journal.pone.0162723)
Supplement: S1 Table — (DOCX) [file pone.0162723.s005.docx]

**Table S1. Elemental analysis of VP2.51**

| Element | Calculated for (C_15_H_17_N_3_O_3_S) | Found |
| --- | --- | --- |
| C | 56.41 | 56.70 |
| H | 5.37 | 5.61 |
| N | 13.16 | 13.22 |
| S | 10.04 | 10.16 |
