# Supplementary material for: Small GSK-3 Inhibitor Shows Efficacy in a Motor Neuron Disease Murine Model Modulating Autophagy
Source: PLoS One. 2016 Sep 15;11(9):e0162723. doi: 10.1371/journal.pone.0162723 (PMC5025054; doi:10.1371/journal.pone.0162723)
Supplement: S2 Table — (DOCX) [file pone.0162723.s006.docx]

**Table S2.** Permeability in the PAMPA-BBB assay for 10 commercial drugs and VP2.51 with its predictive penetration in the CNS.^a^

| Compound | Bibl.^b^ | *Pe* (10^-6^ cm s^-1^)^c^ | BBB prediction |
| --- | --- | --- | --- |
| Atenolol | 0.8 | 0.5 ± 0.3 |  |
| Caffeine | 1.3 | 1.6 ± 0.2 |  |
| Desipramine | 12 | 14.9 ± 1.1 |  |
| Enoxacin | 0.9 | 0.9 ± 0.3 |  |
| Hydrocortisone | 1.9 | 2.2 ± 0.3 |  |
| Ofloxacin | 0.8 | 1.2 ± 0.2 |  |
| Piroxicam | 2.5 | 1.7 ± 0.3 |  |
| Promazine | 8.8 | 8.9 ± 1.0 |  |
| Testosterone | 17 | 15.9 ± 1.1 |  |
| Verapamil | 16 | 16.0 ± 0.8 |  |
| **VP2.51** |  | 7.6 ± 0.4 | CNS + |

^a^ PBS:DMSO (95:5) was used as solvent. ^b^ Reference^[[1]](#footnote-1)^. ^c^ Data are the mean ± SD of 3 independent experiments.

1. Di, L.; Kerns, E. H.; Fan, K.; McConnell, O. J.; Carter, G. T. High throughput artificial membrane permeability assay for blood-brain barrier. *Eur. J. Med. Chem.* **2003,** *38*, 223-232 [↑](#footnote-ref-1)
